# Gut Microbiota and Dopamine: Producers, Consumers, Enzymatic Mechanisms, and In Vivo Insights

**DOI:** 10.3390/bioengineering13010055

**Published:** 2025-12-31

**Authors:** Giovanni Albani, Vasuki Ranjani Chellamuthu, Lea Morlacchi, Federica Zirone, Maryam Youssefi, Marica Giardini, Yin-Xia Chao, Eng-King Tan, Salvatore Albani

**Affiliations:** 1 Division of Neurorehabilitation of Veruno Institute, Istituti Clinici Scientifici Maugeri IRCCS, 28013 Gattico-Veruno, Piedmont, Italy; g.albani@auxologico.it (G.A.); lea.morlacchi@gmail.com (L.M.); marica.giardini@icsmaugeri.it (M.G.); 2 Translational Immunology Institute, SingHealth/Duke-NUS Academic Medical Centre, Singapore 169856, Singapore; 3 Department of Industrial and Information Engineering, Biomedical Engineering, University of Pavia, 27100 Pavia, Italy; 4 Department of Biosciences, Science and Technology, University of Milan, 20133 Milan, Italy; 5 Duke-National University of Singapore Medical School, Singapore 169857, Singapore; 6 National Neuroscience Institute, Singapore 308433, Singapore; 7 Department of Neurology, Singapore General Hospital, Singapore 169608, Singapore

**Keywords:** gut microbiota, dopamine, tyrosine decarboxylase (TDC), aromatic L-aminoacid decarboxylase (AADC), L-DOPA

## Abstract

The human gut microbiota plays a key role in neurochemical communication, especially through the gut–brain axis. There is growing evidence that the gut microbiota influences dopamine metabolism through both production and consumption mechanisms. Two key bacterial enzymes are central to this process: tyrosine decarboxylase (TDC), which primarily catalyzes the decarboxylation of tyrosine to tyramine but can also act on L-DOPA to produce dopamine in certain bacterial strains, and aromatic L-amino acid decarboxylase (AADC), which can convert precursors such as L-DOPA, tryptophan, or 5-hydroxytryptophan into bioactive amines including dopamine, tryptamine, and serotonin. Identifying the bacterial families corresponding to TDC and AADC enzymes opens new avenues for clinical intervention, particularly in neuropsychiatric and neurodegenerative disorders, such as Parkinson’s disease. Moreover, elucidating strain-specific microbial contribution and host-microbe interactions may enable personalized therapeutic strategies, such as selective microbial enzyme inhibitors or tailored probiotics, to optimize dopamine metabolism. Emerging technologies, including biosensors and organ-on-chip platforms, offer new tools to monitor and manipulate microbial dopamine activity. This article explores the bacterial taxa capable of producing or consuming dopamine, focusing on the enzymatic mechanisms involved and the methodologies available for studying these processes in vivo.

## 1. Introduction

The gut microbiota has emerged as a potential regulator of certain brain functions. There is increasing evidence showing that intestinal microbes can synthesize, metabolize, or modulate neurotransmitters, including dopamine, thereby influencing mood, cognition, and behavior. Alterations in microbial composition have also been associated with neuropsychiatric and neurodegenerative disorders, suggesting a bidirectional “gut–brain axis” [1]. Specific gut commensal bacteria can produce bioactive amines capable of modulating dopaminergic signaling pathways, highlighting the therapeutic potential of microbiota-targeted interventions in Parkinson’s disease (PD) and depression [2,3,4,5].

Experimental models and fecal microbiota transplants (FMT) have been pivotal in exploring gut–brain communication. Animal studies demonstrate that germ-free mice exhibit altered stress responses, cognition, and dopaminergic signaling, effects that can be partially restored through FMT from healthy donors. Similarly, transplantation of microbiota from patients with neurological or psychiatric disorders can transfer behavioral and metabolic traits, underscoring a causal role of the gut microbiota in brain function. These findings highlight the therapeutic potential of microbiota-targeted interventions for neurodegenerative and mood disorders, although robust clinical trials are still needed [2,3].

Human studies are fewer but expanding, yet they provide important insights into the role of gut microbiota in dopaminergic regulation. Several investigations have associated distinct microbial profiles with altered blood dopamine levels, impulsivity, or neuropsychiatric symptoms, supporting the translational relevance of preclinical findings. Moreover, recent research has identified bacterial enzymes capable of converting levodopa into dopamine in the gut, thereby limiting its central availability. Pharmacological inhibition of these microbial enzymes has emerged as a promising strategy to improve therapeutic efficacy in PD and to fine-tune host dopamine homeostasis [6,7].

The distinction between gut bacteria that produce dopamine and those that consume or metabolize dopamine is crucial to understanding the neurochemical balance of the gut and its possible neurological implications (e.g., in PD or behavior).

Building on these insights, advances in sequencing technologies now allow an unbiased exploration of microbial contributions to dopamine metabolism [8]. Yet such technologies alone cannot capture dynamic host-microbe interactions, making in vivo evaluation a critical next step. Integrating genomic insights with in vivo evaluation is essential to confirm functional activity and understand the physiological impact.

## 2. Dopamine Synthesis and Metabolism in Humans and the Gut Microbiota

Dopamine is a key neurotransmitter in motor control, cognition, and reward. In humans, dopamine biosynthesis begins with the amino acid L-tyrosine [9]. The first step is the hydroxylation of L-tyrosine to L-DOPA (L-3,4-dihydroxyphenylalanine), catalyzed by tyrosine hydroxylase (TH), the rate-limiting enzyme of the pathway, with tetrahydrobiopterin (BH4) as a cofactor. In the second step, AADC, also known as DOPA decarboxylase (DDC), converts L-DOPA into dopamine using pyridoxal phosphate (vitamin B6) as a cofactor. This process occurs mainly in dopaminergic neurons of the substantia nigra pars compacta and ventral tegmental area, but dopamine synthesis also takes place in peripheral tissues such as the kidney and gastrointestinal tract.

Dopamine metabolism occurs across several interconnected anatomical and biochemical compartments that collectively regulate its synthesis, storage, signaling, and degradation [10,11]. In the presynaptic neuron, dopamine is synthesized from the amino acid tyrosine via TH and AADC, and subsequently stored in synaptic vesicles through VMAT2, which protects it from cytosolic degradation [10,12]. Upon neuronal activation, dopamine is released into the synaptic cleft, where it binds to D1-like and D2-like receptors on postsynaptic neurons before its actions are terminated primarily by reuptake through the dopamine transporter (DAT), returning dopamine to the presynaptic terminal for recycling [12]. Glial cells, particularly astrocytes, also take up dopamine and contribute to its enzymatic degradation via monoamine oxidase (MAO) and catechol-O-methyltransferase (COMT), yielding metabolites such as DOPAC and HVA [10,11]. Cytosolic dopamine that escapes vesicular storage is prone to oxidation, generating reactive oxygen species and contributing to selective dopaminergic vulnerability, especially relevant in Parkinson’s disease (PD) [11]. Beyond the central nervous system, dopamine metabolism also occurs in peripheral compartments including the gastrointestinal tract and kidneys, where dopamine acts locally and remains largely independent from central pools due to the blood–brain barrier [11,13].

Beyond its basic biochemistry, dopamine metabolism is regulated by multiple endogenous and environmental factors that influence its synthesis, degradation, receptor availability, and signaling efficiency. Genetic variability plays a central role, with polymorphisms in key genes such as *COMT* (catechol-O-methyltransferase that degrades dopamine), *MAO-A/B* (monoamine oxidase that metabolize dopamine), *DRD2/3/4* (dopamine receptor genes), the dopamine transporter DAT (*SLC6A3*), and *TH* encoding tyrosine hydroxylase (the rate limiting enzyme in dopamine synthesis) altering dopaminergic tone [14,15,16]. Lifestyle factors such as physical activity (increases dopamine release and up regulates dopamine receptors), sleep deprivation (reduction in dopamine receptor availability and disrupts synthesis), stress (elevates cortisol that can impair dopamine pathways over time), overstimulation from modern behaviors (e.g., excessive gaming or social media use) and finally meditation and relaxation (normalizes dopamine tone) can modulate dopaminergic tone and hypothalamic–pituitary–adrenal (HPA) axis regulation [17,18]. Dietary influences are also significant, as foods rich in tyrosine and phenylalanine (e.g., meat, dairy, soy, nuts) enhance precursor availability for dopamine synthesis [19], whereas high-fat diets may disrupt dopaminergic and opioid signaling across development [20]. Aging introduces additional complexity through neurovascular alterations and reduced dopaminergic function, which may increase susceptibility to neurodegenerative processes [21]. Finally, pharmacological agents such as L-DOPA, methylphenidate, amphetamines, MAO inhibitors, cocaine, and antipsychotics exert direct effects on dopamine synthesis, degradation, receptor binding, or transporter activity [22,23,24]. Collectively, these genetic, lifestyle, nutritional, pharmacological, and aging-related determinants contribute to interindividual variability in dopamine homeostasis and modulate vulnerability to neurological and psychiatric disorders.

Beyond host tissues, however, dopamine metabolism is increasingly recognized to be shaped by the gut microbiota, which harbors enzymes capable of modulating this pathway. Understanding these microbial contributions is critical as they directly influence both physiological regulation and therapeutic outcomes.

Genomic studies have revealed the widespread presence of decarboxylase enzymes in gut bacteria [6]. These include TDC and AADC, which are capable of catalyzing the decarboxylation of L-DOPA to dopamine. Homologous AADC-like enzymes have been detected in *Clostridium sporogenes* and *Lactobacillus brevis*, with additional evidence in *Enterococcus* and *Escherichia coli*. Unlike humans, many gut bacteria lack TH and instead rely on TDC, which primarily converts L-tyrosine into tyramine, a neuromodulatory compound (Table 1-[6,7,25,26,27,28,29,30,31,32,33]).

Importantly, TDC shows a dual potential. This pyridoxal phosphate-dependent enzyme, enriched in *Enterococcus faecalis* and *Enterococcus faecium*, normally converts tyrosine to tyramine but can also decarboxylate L-DOPA to dopamine.

**Figure 1:**
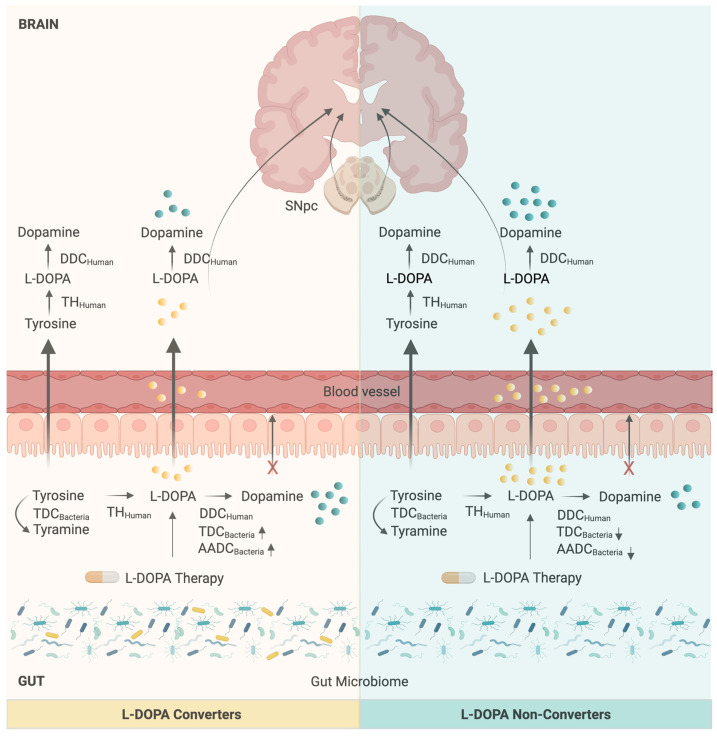
Host and gut microbiome contribution to dopamine metabolism. In humans, dopamine is synthesized from tyrosine via tyrosine hydroxylase (TH_Human_) and dopa decarboxylase (DDC_Human_), with L-DOPA serving as the intermediate that crosses the blood–brain barrier (BBB). In the gut, microbial tyrosine decarboxylase (TDC_Bacteria_) converts tyrosine to tyramine, while both human DOPA decarboxylase (DDC_Human_) and microbial decarboxylases (TDC_Bacteria_/AADC_Bacteria_) in certain bacteria (L-DOPA converters) can convert L-DOPA into dopamine. However, peripherally produced dopamine cannot cross the BBB, reducing systemic L-DOPA availability for central dopamine synthesis. Created with BioRender.com.

Clinically, this is highly relevant for PD patients treated with L-DOPA. L-DOPA (levodopa) is a precursor of dopamine that crosses the blood–brain barrier, where it is converted into dopamine, thereby replacing the reduced dopamine level in PD and other neurological disorder patients and alleviating their motor symptoms. However, L-DOPA can also be metabolized by bacterial TDC in the gut, which result in reduced therapeutic efficacy and more peripheral side effects (Figure 1). L-DOPA is always administered in combination with benserazide or carbidopa, a peripheral AADC inhibitor. Carbidopa prevents the conversion of L-DOPA to dopamine outside the brain, allowing more L-DOPA to reach the CNS and reducing peripheral side effects. However, unlike human AADC, microbial TDC is not inhibited by carbidopa, meaning gut bacteria can effectively “steal” the drug [6,34]. Excitingly, encouraging recent work has shown that targeted inhibitors of bacterial TDC can block this unwanted metabolism and restore L-DOPA availability [6].

## 3. Dopamine-Producing and Consuming Bacterial Families

While dopamine generated in the gut cannot cross the blood–brain barrier, it plays important local roles. Microbial AADC activity contributes to the pool of dopamine in the intestinal lumen, influencing motility, secretion, and mucosal signaling through the enteric nervous system, thereby linking microbial metabolism to host physiology [5,35].

Several bacterial genera, such as *Bacteroides*, *Clostridium*, and *Enterococcus*, have been implicated in dopamine metabolism, with evidence supporting dopamine production via AADC activity in some cases. Others, such as *Coprococcus eutactus* (Lachnospiraceae), are associated with enhanced dopaminergic tone without directly decarboxylating L-DOPA (Table 2). Conversely, bacteria expressing TDC, such as *Enterococcus faecalis*, can metabolize L-DOPA, reduce its systemic availability and potentially influence dopamine-based therapies. Certain species, like *Alistipes indistinctus*, may indirectly lower dopamine levels by altering the metabolism of precursor amino acids such as tyrosine [5,36].

Other bacterial genera, including *Clostridium* spp. and *Ruminococcus* spp., are also involved in the synthesis of neurotransmitters and regulation of the gut–brain axis. These bacteria may influence dopamine availability in the gut, thereby potentially affecting the central nervous system through the gut–brain axis. While these taxa are associated with dopaminergic signaling, the specific microbial genes and pathways underlying their effects on dopamine metabolism remain incompletely defined. Unbiased, genome-resolved approaches are therefore essential to systematically identify microbial contributors to dopamine-related metabolic potential within the gut microbiome.

## 4. Shotgun Sequencing

Shotgun sequencing, and, in particular, shotgun metagenomics, is a powerful high-throughput method used to study the composition and functional potential of complex microbial communities. Unlike targeted sequencing approaches such as 16S rRNA amplicon sequencing, shotgun methods randomly fragment the entire DNA content of a sample and sequence all fragments in parallel. This generates a comprehensive and unbiased dataset covering bacteria, archaea, fungi, viruses, and even host DNA when present [37,38].

One of the main advantages of shotgun sequencing is that it provides both taxonomic resolution at the species or even strain level and functional insights into the metabolic pathways encoded by the community [39]. This makes it particularly valuable in studies where host-microbe interactions or microbial enzymatic activities are of interest, such as in the gut microbiota’s role in neurological and metabolic diseases [40].

## 5. In Vivo and Translational Evaluation

Currently, there are no standardized clinical devices to specifically detect the presence of dopamine-producing bacterial families in the gut. However, several promising technologies in this area are being explored by scientific research.

**Modulation of intestinal dopamine metabolism**

A promising approach involves the selective inhibition of bacterial decarboxylases to modulate intestinal dopamine without interfering with human enzymes, thereby optimizing dopaminergic therapies. This is exemplified by the finding that bacterial TDC enzyme from *Enterococcus faecalis* converts L-DOPA to dopamine in the gastrointestinal tract, thereby reducing the drug’s cerebral availability. Notably, selective inhibition with compounds such as (S)-α-fluoromethyltyrosine (AFMT), are effectively capable of blocking bacterial decarboxylation of L-DOPA without interfering with human decarboxylation, thus increasing the drug’s bioavailability [6].

**Electrochemical biosensors**

Electrochemical biosensors are capable of detecting dopamine through electrochemical reactions. Although they have been primarily used to measure dopamine in body fluids such as blood or saliva, their application for direct monitoring in the intestinal tract is still under development [41,42,43,44,45,46].

**Aptamer-based biosensors** use DNA or RNA molecules that can specifically bind to certain targets, such as dopamine. These biosensors offer high specificity and sensitivity in the detection of neurotransmitters. However, their application to monitor dopamine production by the gut microbiota is still under investigation [47,48,49,50].

**Ingestible biosensors** are smart capsules designed to monitor the gut environment in real time. These devices can detect specific metabolites, such as dopamine, directly in the gastrointestinal tract. For example, a recent study developed an ingestible biosensor that uses engineered bacteria to record the presence of gut metabolites, providing valuable data on the production of neurotransmitters by the microbiota [51,52,53,54].

**Figure 2:**
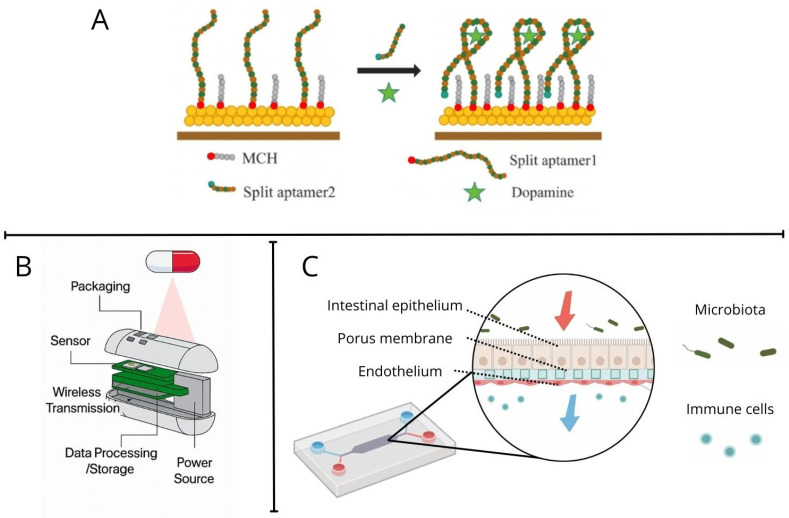
Emerging platforms for studying dopamine detection and host-microbe interactions in the gut. (**A**) Schematic representation of an amperometric split-aptamer biosensor for dopamine detection. One aptamer strand is immobilized on a gold electrode, while the complementary strand is labeled with a methylene blue (MB) redox reporter. The electrode surface is blocked with mercaptohexanol (MCH) to minimize nonspecific interactions. Dopamine binding induces formation of a folded sandwich complex (aptamer 1–dopamine–aptamer 2), facilitating electron transfer and signal generation. (**B**) Ingestible biosensors designed as smart capsules for real-time monitoring of the gastrointestinal environment. These devices enable direct, in situ detection of metabolites such as dopamine within the gut. (**C**) Schematic of a two-channel gut-on-chip platform separated by a porous membrane. The upper microchannel hosts intestinal epithelial cells, while the lower channel accommodates other cell types, typically vascular endothelial cells. Controlled apical and basolateral flows support coculture with microbiota and immune components, enabling physiologically relevant modeling of host-microbe interactions.

While emerging in vivo biosensors and ingestible devices hold promise for the real-time monitoring of microbial dopamine activity, they cannot fully recapitulate the complexity of human intestinal pathology. In particular, the integration of microbial enzymatic activity with host epithelial, immune, vascular and neuronal components remains difficult to resolve in vivo. Figure 2 provides an overview of representative in vitro, in vivo applicable, and organ-on-chip platforms currently employed or under development to study dopamine sensing and host-microbe interactions in the gut [51,55,56]. Together, these limitations highlight the need for complementary platforms such as organ-on-chip technologies, which offer controlled yet physiologically relevant environments to study host-microbe enzymatic interactions.

## 6. Organ on Chip: A Platform to Model Host-Microbe Dopamine Interactions

Bacterial and host enzymes shape dopamine metabolism, but studying their interactions in vivo is challenging due to interspecies differences, complex microbial ecosystems, and ethical constraints. Also, sampling intestinal contents and mucosal enzymes in humans is restrictive, so much of the human-microbiome biology remains hidden.

Organ-on-chip technologies have emerged as advanced microphysiological platforms capable of recapitulating essential features of the human intestinal microenvironment within a controlled microfluidic platform [57,58,59]. Contemporary gut-on-chip models integrate polarized intestinal epithelial cells with endothelial and immune components under dynamic flow and mechanical stimulation, enabling more physiologically relevant modeling of host-microbe interactions [59,60,61]. Importantly, these platforms allow controlled co-culture of defined microbial communities, overcoming some of the variability inherent in in vivo studies [62].

From the perspective of dopamine biology, organ-on-chip systems could enable study of the spatial and temporal interplay between microbial enzymes and host enzymes such as TDC, AADC (aromatic L-amino acid decarboxylase), COMT (catechol O-methyltransferase), and MAO (monoamine oxidase), which influence dopamine availability, including platforms that promote maturation of human iPSC-derived dopaminergic neurons for PD modeling [63]. Such a system will also facilitate therapeutic testing such as selective microbial enzymatic inhibitors, dietary modulators, and engineered probiotics, under human relevant conditions.

Next-generation gut–brain-on-chip platforms integrate neuronal compartments with real-time biosensors to track exosome trafficking and metabolite flux across barrier, capturing dynamic gut-derived signaling inaccessible in static assays [64,65]. Further recent advances have extended these systems toward multi-organ configurations incorporating gut–brain models, enabling investigation of metabolite and neurotoxin transport from the intestine to neural compartments [66,67]. These developments are especially relevant for PD, where peripheral dopamine metabolism and microbial activity may influence central pathology [68].

Despite their promise, current organ-on-chip platforms face significant limitations. Most systems rely on simplified microbial consortia that do not fully capture the diversity of the human gut microbiota [69]. Integration of enteric neurons and long-term immune-microbial interactions remains technically challenging, and real-time quantification of low-abundance metabolites such as dopamine and L-DOPA requires further development of embedded biosensors. Additionally, variability in chip design, cell sourcing, and culture conditions hampers cross-study comparability and standardization.

Future progress in this field will depend on the incorporation of multi-layered sensing technologies, improved modeling of the enteric nervous system, and standardized frameworks that enable reproducible interrogation of microbial-host dopamine metabolism across platforms. Addressing these challenges will be essential for translating organ-on-chip insights into clinically relevant strategies for modulating levodopa bioavailability and dopaminergic signaling in PD.

## 7. Bifidobacterium and Certain Lactobacillus Strains as Potential Candidates for Parkinson’s Disease

*Bifidobacterium* species are Gram-positive, anaerobic, saccharolytic bacteria that represent key members of the healthy human gut microbiota, particularly in early life. They are known to participate in amino acid metabolism, including pathways involving aromatic amino acids such as tyrosine and tryptophan. However, unlike *Enterococcus faecalis*, *Bifidobacterium* spp. do not possess the TDC enzyme responsible for decarboxylating L-DOPA to dopamine [6]. Instead, several *Bifidobacterium* strains metabolize L-DOPA through an oxidative-reductive pathway, converting it into 3,4-dihydroxyphenyl-lactic acid (DHPLA) via aromatic lactate dehydrogenases, thereby preventing dopamine accumulation [70]. DHPLA is a metabolic dead end for L-DOPA: once formed, it is not converted back. This pathway can be quantified by in vitro batch cultures and enzyme kinetics. However, the effect compared to TDC is very small; for example, *Enterococcus faecalis* harboring TDC can decarboxylate ~100% of 1 mM L-DOPA to dopamine within 24 h in culture, while *Bifidobacterium* species, under similar in vitro conditions, convert only ~1–3% in rich medium and ~12% in minimal medium to DHPLA after 48 h [70]. Thus, based on the current evidence, the DHPLA pathway may function as a microbial ecology signal, reflecting growth on residual L-DOPA in the colon.

This reaction is considered detoxifying and may modulate local redox balance and intestinal barrier integrity. Moreover, certain *Bifidobacterium* contributes indirectly to neurotransmitter homeostasis by producing γ-aminobutyric acid (GABA) and influencing host tryptophan-indole metabolism [71]. Current evidence therefore indicates that *Bifidobacterium* spp. are not L-DOPA-decarboxylating bacteria and may even protect levodopa bioavailability by shunting it away from dopaminergic degradation pathways. Their presence in the Parkinsonian gut is often reduced, suggesting a potential role in maintaining a metabolic environment favorable to therapeutic L-DOPA absorption.

*Lactobacillus reuteri* is a Gram-positive, facultatively heterofermentative bacterium widely recognized for its probiotic and immunomodulatory properties. It participates in the metabolism of amino acids and biogenic amines through the activity of AADC, particularly TDC, which can convert tyrosine to tyramine [72]. However, the ability of *L. reuteri* to decarboxylate L-DOPA to dopamine remains uncertain and likely limited. While some *Lactobacillus* species such as *L. brevis* possess TDC variants capable of acting on L-DOPA in vitro, there is no robust evidence that *L. reuteri* contributes significantly to L-DOPA degradation or interferes with dopaminergic bioavailability in vivo [6,7,73]. Instead, *L. reuteri* appears to exert indirect neuromodulatory effects by producing γ-aminobutyric acid (GABA) via glutamate decarboxylase and modulating host tryptophan-indole pathways, thereby influencing gut–brain communication [74]. Overall, current data suggest that *L. reuteri* participates in amine metabolism through tyramine and GABA synthesis but does not play a direct role in intestinal L-DOPA decarboxylation, differentiating it from strongly L-DOPA-metabolizing taxa such as *Enterococcus faecalis*.

Within the broader context of the microbiota-gut–brain axis, *Bifidobacterium* and related lactic acid bacteria have been implicated in shaping host dopaminergic signaling and metabolism, highlighting their potential relevance despite limited direct interaction with L-DOPA [75].

## 8. Discussion

The interplay between the gut-microbiota and host dopamine metabolism represents a critical interface of neurochemistry, microbiology, and clinical medicine. Recent studies underscore that the gut microbiota is not merely a passive community but an active contributor to the intestinal catecholamine pool, influencing both local and systemic physiology. Advances in sequencing, metabolomics, and organ-on-chip technologies have now made it possible to characterize the microbial contributions with great resolution, revealing enzymatic mechanisms that were previously hidden.

AADC is the classical enzyme that converts L-DOPA into dopamine in humans, where it is central to catecholamine biosynthesis. Interestingly, the human microbiota can also synthesize dopamine or modulate its precursors. A subset of gut microbes, such as *Enterococcus faecalis*, can metabolize L-DOPA into dopamine via bacterial decarboxylases that differ from human AADC. This microbial activity has clinical implications, as it can reduce L-DOPA availability, limiting the effectiveness of PD therapy. Thus, while human dopamine synthesis follows a highly regulated two-step enzymatic pathway, microbial metabolism introduces variability that can profoundly affect host neurochemistry and treatment outcomes. Besides PD, microbial catecholamine metabolism may also have a role in mood and behavior. Gut microbes are now recognized as a significant source of biologically active catecholamines, including dopamine and norepinephrine [76].

When the microbiome becomes imbalanced, these metabolic pathways can shift, contributing to disturbances in amino acid metabolism and behaviors linked to mood disorders, as shown in both human studies and animal models [2]. Together, these findings highlight bacterial AADC and TDC as key enzymes at the intersection of microbiology and medicine, shaping gut–brain communication and influencing clinical outcomes ranging from mental health to the effectiveness of PD therapies.

Beyond microbial dopamine production and consumption, host and environment related factors including aging, lifestyle, diet, and particularly prebiotic intake, critically modulate these processes by shaping gut microbial composition and metabolic activity. Prebiotics, defined as non-digestible dietary fibers that selectively promote the growth and functional persistence of beneficial gut bacteria, are increasingly recognized as indirect regulators of dopaminergic homeostasis along the gut–brain axis [77]. By enriching microbial taxa involved in short-chain fatty acid (SCFA) production (acetate, propionate, and butyrate), prebiotics improve intestinal barrier integrity, reduce systemic inflammation, and influence the bioavailability of dopamine precursors such as tyrosine and phenylalanine [78,79].

Although intestinal dopamine itself cannot cross the blood–brain barrier, these downstream effects could potentially support central dopaminergic signaling by limiting neuroinflammatory tone and microglial activation. Thus, prebiotics act not as direct neuromodulators, but as ecological drivers that influence microbial metabolism and host inflammatory state, thereby indirectly shaping dopamine related pathways.

One of the key points of this work is to underline that the influence of intestinal bacteria on dopaminergic function likely occurs only at the peripheral level, as there is currently no evidence that intestinally produced dopamine can cross the blood–brain barrier (BBB) [80]. Indeed, dopamine is a relatively polar catecholamine. Because of its hydrophilic structure (two hydroxyl groups on the benzene ring and an amine group), it cannot easily diffuse across the lipid bilayer of endothelial cells in the BBB. The BBB has selective transporters for certain molecules (e.g., amino acids, glucose, and some neurotransmitter precursors) [81]. However, it lacks a dedicated transporter for dopamine. Instead, the brain imports L-DOPA, the metabolic precursor of dopamine, via the large neutral amino acid transporter (LAT1). Once inside the brain, L-DOPA is converted into dopamine by AADC [82]. This explains why in PD therapy, patients are given L-DOPA, rather than dopamine itself. Administered dopamine would remain in the periphery, acting mainly on the cardiovascular system, without reaching the central nervous system.

In this context, it is therefore necessary to take into account the intestinal bacteria capable of influencing the absorption of L-DOPA taken as a drug, and the metabolism of dopamine obtained from dietary sources (e.g., bananas) [83], or dopamine produced internally within the host. Among these, certain probiotic genera like Lactobacillus warrant particular attention as they represent the most widely marketed species globally and demonstrate dopamine-producing capabilities.

Lactobacillus strains such as *Lactobacillus brevis* and *Lactobacillus plantarum* are capable of producing dopamine and other neurotransmitters in vitro and in animal models, where this has been associated with behavioral modulation and changes in plasma dopamine levels [75]. It is plausible that these strains also produce dopamine in the human gut, although the amount produced is likely much lower than central neuronal dopamine. While intestinal dopamine does not directly cross the blood–brain barrier, it can influence the central nervous system indirectly via the gut–brain axis, including the vagus nerve, immune signaling, microbial metabolites, and modulation of precursors such as L-DOPA [75].

On the other hand, *Lactobacillus reuteri* appears unlikely to participate in L-DOPA conversion. Although TDC genes are prevalent in *Enterococcus* and in certain *Lactobacillus* species (e.g., *L. brevis*), there is no compelling evidence that *L. reuteri* decarboxylates L-DOPA to dopamine in vivo or in vitro under physiologically relevant conditions [84]. By comparison, *Bifidobacterium* spp. also lacks the ability to decarboxylate L-DOPA to dopamine. Recent studies indicate that some *Bifidobacterium* species metabolize L-DOPA through an alternative route, converting it to 3,4-dihydroxyphenyl-lactate (DHPLA) via tyrosine-metabolizing enzymes; therefore, they do not increase luminal dopamine from L-DOPA [70]. Overall, most evidence attributes the microbial conversion of L-DOPA to dopamine to *Enterococcus faecalis* (via TDC activity), followed by the subsequent dopamine to tyramine step mediated by *Eggerthella lenta* [6].

In parallel with advances in mechanistic understanding, a growing body of work has explored microbiome-directed interventions for PD. These include probiotics, prebiotics, synbiotics, postbiotics, and FMT, all of which aim to modulate gut microbial composition or metabolic output [85]. Probiotics show the most consistent benefit for gastrointestinal motility and selected non-motor symptoms, while effects on motor function (MDS-UPDRS III) remain small and heterogeneous across trials [86]. Prebiotics, particularly fermentable fibers such as resistant starch, appear to enhance SCFA production and reduce inflammatory markers, and may attenuate non-motor symptom burden, though current clinical data is derived from small, predominantly open-label studies [87,88]. Synbiotic combinations may confer broader effects on oxidative stress and mental health compared with probiotics alone, but evidence remains limited by small sample sizes and heterogeneity in formulations [89]. FMT, while biologically compelling, remains experimental; outcomes depend heavily on donor selection, dosing strategy, and disease stage. Although several observational studies suggested benefit, a recent randomized clinical trial reported neutral effects, underscoring the need for standardized protocols and larger trials before routine clinical adoption [90]. Overall, these interventions illustrate promising but still preliminary translational avenues, reinforcing the central role of the gut microbiota in PD pathophysiology while highlighting the need for rigorous mechanistic and clinical validation.

In the future, integrating multi-omics analyses with in vivo-like organ-on-chip studies will be critical to fully elucidate how microbial enzymatic activity modulates host dopamine physiology. Emerging biosensors and ingestible device offer potential for real-time monitoring of intestinal dopamine, providing translational tools to personalize therapies and outcomes. Understanding, strain specific microbial contribution and the individualized dynamics of host-microbe interactions could pave the way for personalized interventions, such as selective microbial enzyme inhibitors or tailored probiotic formulations, aimed at optimizing intestinal dopamine metabolism to improve neurological outcomes.

## Figures and Tables

**Table 1:** Gut bacterial families with demonstrated or putative involvement in L-3,4-dihydroxyphenylalanine (L-DOPA) metabolism.

Category	Bacterial Family	Evidence for L-DOPA Decarboxylation	Main Enzyme	Genera/Species	Study Type
Consistent Decarboxylators	Enterococcaceae	🟢 Strong	TDC	*Enterococcus faecalis*, *Enterococcus faecium*	In vivo (human), in vitro
Strain-Dependent /Occasional	Streptococcaceae	🟡 Moderate	TDC	*Streptococcus thermophilus*	In vivo (animal), in vitro
	Lactobacillaceae	🟡 Moderate	TDC	*Lactobacillus brevis*, *L. curvatus*, *L. fermentum*	In vivo (human and animal), in vitro
		🔴 Negative	–	*L. reuteri*	In vivo (human), in vitro
	Clostridiaceae	🟡 Moderate	TDC or AADC	*Clostridium sporogenes*, *C. perfringens*	In vitro
	Enterobacteriaceae	🟡 Moderate	AADC	*Escherichia coli, Klebsiella pneumoniae, Enterobacter cloacae*	In vivo (human and animal), in vitro
	Bacillaceae	🟠 Weak/unclear	TDC	*Bacillus subtilis*, *Bacillus cereus*	In vitro
	Lachnospiraceae	🟠 Weak/unclear	AADC in rare cases	*Blautia obeum*, *Roseburia intestinalis*, *Coprococcus eutactus*	In vivo (human)
No Evidence /Negative	Eggerthellaceae	🔴 None	–	*Eggerthella lenta*	In vivo (animal), in vitro
	Bacteroidaceae	🔴 Negative	–	*Bacteroides fragilis*, *B. thetaiotaomicron*	In vivo (animal)
	Ruminococcaceae	🔴 None	–	*Ruminococcus bromii*, *Faecalibacterium prausnitzii*	In vivo (animal), in vitro
	Rikenellaceae	🔴 None	–	*Alistipes putredinis*	In vivo (human)
	Bifidobacteriaceae	🔴 None	–	*Bifidobacterium longum*, *B. breve*	In vivo (human and animal)
	Akkermansiaceae	🔴 None	–	*Akkermansia muciniphila*	In vivo (human and animal), in vitro

The table summarizes bacterial families and representative species reported to engage in L-DOPA decarboxylation through tyrosine decarboxylase (TDC) or aromatic L-amino acid decarboxylase (AADC) pathways. Legend 🟢 Strong evidence: multiple consistent experimental studies demonstrating L-DOPA decarboxylation. 🟡 Some/strain-dependent evidence: activity observed only in specific strains or mainly in vitro. 🟠 Weak/unclear evidence: limited, indirect, or inconsistent data. 🔴 No evidence/negative: no convincing reports of L-DOPA decarboxylation or negative for L-DOPA decarboxylation.

**Table 2:** Indirect modulators of dopaminergic tone.

Bacteria Genera/Species (Family)	Mechanism
*Coprococcus eutactus* (Lachnospiraceae)	SCFA production (butyrate), vagus nerve signaling.
*Alistipes indistinctus* (Rikenellaceae)	Unknown, possibly SCFAs or immune modulation.
*Ruminococcus bromii* (Ruminococcaceae), *Bifidobacterium longum* (Bifidobacteriaceae), *Blautia obeum* (Lachnospiraceae)	SCFAs, tryptophan/kynurenine metabolism.

## Data Availability

No new data were created or analyzed in this study.
